# Simultaneous Quantitative SARS-CoV-2 Antigen and Host Antibody Detection and Pre-Screening Strategy at the Point of Care

**DOI:** 10.3390/bioengineering10060670

**Published:** 2023-06-01

**Authors:** Kritika Srinivasan Rajsri, Michael P. McRae, Nicolaos J. Christodoulides, Isaac Dapkins, Glennon W. Simmons, Hanover Matz, Helen Dooley, David Fenyö, John T. McDevitt

**Affiliations:** 1 Division of Biomaterials, Department of Molecular Pathobiology, New York University School of Dentistry, New York, NY 10010, USA; kritika.srinivasanks3144@nyu.edu (K.S.R.); michael.mcrae@nyu.edu (M.P.M.); nicolaoschristo19@gmail.com (N.J.C.); gwsimmons@gmail.com (G.W.S.); 2 Department of Pathology, Vilcek Institute of Graduate Biomedical Sciences, New York University School of Medicine, New York, NY 10010, USA; 3 Departments of Population Health and Medicine, New York University School of Medicine, New York, NY 10010, USA; isaac.dapkins@nyulangone.org; 4 Department of Microbiology and Immunology, University of Maryland School of Medicine, Baltimore, MD 21201, USA; hmatz@som.umaryland.edu (H.M.); hdooley@som.umaryland.edu (H.D.); 5 Department of Biochemistry and Molecular Pharmacology, New York University School of Medicine, New York, NY 10010, USA; david@fenyolab.org

**Keywords:** artificial intelligence, COVID-19, clinical decision making, lab-on-a-chip, nucleocapsid antigen, spike RBD IgG, SARS-CoV-2 immunity

## Abstract

As COVID-19 pandemic public health measures are easing globally, the emergence of new SARS-CoV-2 strains continue to present high risk for vulnerable populations. The antibody-mediated protection acquired from vaccination and/or infection is seen to wane over time and the immunocompromised populations can no longer expect benefit from monoclonal antibody prophylaxis. Hence, there is a need to monitor new variants and its effect on vaccine performance. In this context, surveillance of new SARS-CoV-2 infections and serology testing are gaining consensus for use as screening methods, especially for at-risk groups. Here, we described an improved COVID-19 screening strategy, comprising predictive algorithms and concurrent, rapid, accurate, and quantitative SARS-CoV-2 antigen and host antibody testing strategy, at point of care (POC). We conducted a retrospective analysis of 2553 pre- and asymptomatic patients who were tested for SARS-CoV-2 by RT-PCR. The pre-screening model had an AUC (CI) of 0.76 (0.73–0.78). Despite being the default method for screening, body temperature had lower AUC (0.52 [0.49–0.55]) compared to case incidence rate (0.65 [0.62–0.68]). POC assays for SARS-CoV-2 nucleocapsid protein (NP) and spike (S) receptor binding domain (RBD) IgG antibody showed promising preliminary results, demonstrating a convenient, rapid (<20 min), quantitative, and sensitive (ng/mL) antigen/antibody assay. This integrated pre-screening model and simultaneous antigen/antibody approach may significantly improve accuracy of COVID-19 infection and host immunity screening, helping address unmet needs for monitoring vaccine effectiveness and severe disease surveillance.

## 1. Introduction

As the fight against COVID-19 enters its fourth year, public health mandates around the pandemic started moving towards surveillance of new variants of concern (VOC), vaccination efficacy, and protection of vulnerable populations. While over 13 billion vaccine doses were administered worldwide so far [1], studies showed that antibody-mediated protection wanes over time and is variable in certain populations (aged and/or immunocompromised individuals, those with underlying medical conditions, etc.) [2,3]. Furthermore, the SARS-CoV-2 virus continues to evolve. Indeed, the Omicron VOC was the dominant driver for global disease spread since 2022, having high transmission and infectivity rates, a propensity for break-through infections, and evasion of vaccination-induced immunity [4,5,6]. This situation is a concern for the at-risk populations—older adults, individuals with certain underlying medical conditions, and the immunocompromised—where the rates of severe disease and hospitalization remain high [7,8]. Though the bivalent vaccines showed improved efficacy and protection against the Omicron variant [9,10], only 17% of the total population received the updated vaccination [11]. With easing of containment measures (e.g., masking, social distancing, self-isolation, travel restrictions) [12], waning seroprevalence levels, plus high transmissibility, and infectivity associated with new VOCs, transmission of infection to the at-risk communities is a challenging issue [4,13].

During this new phase of pandemic response, screening and surveillance of the at-risk population can help contain transmission rates, guide vaccination strategy, and aid the prevention of severe disease and associated morbidity and mortality. Additionally, a screening strategy may help guide convalescent plasma donations, a key therapy for immunocompromised patients who are susceptible to refractory infection [14]. With rising transmissibility associated with newer VOCs, waning immunity across population and asymptomatic/pre-symptomatic cases serving as a driving force for the community spread of COVID-19 [15,16,17], population level at-home antigen tests became a critical tool for breaking the chain of transmission [18].

Real-time reverse transcriptase polymerase chain reaction (RT-PCR) remained the current gold standard method for SARS-CoV-2 detection, especially in the first 5 days of infection with viral load peaking around day 4 [19,20]. While this method has excellent sensitivity, results are usually reported in days, and this method requires specialized laboratories and highly trained technicians, making the methodology unsuitable for POC screening. Although potentially less sensitive than RT-PCR, rapid (~15 min) and inexpensive immunoassays for SARS-CoV-2 antigen detect specific viral proteins (e.g., S protein, NP, hemagglutinin esterase protein) found in the virus and are deemed more appropriate for POC use. POC testing strategies utilized for the COVID-19 pandemic response include testing viral nucleic acid, viral antigen, viral protein, host antibody, and cytokines. While RT-PCR and ELISA represent quantitative gold-standard lab based clinical testing, the use of lateral-flow assays, miniaturized PCR, lab-on-a-chip, microfluidics-paper based assays, isothermal nucleic acid testing, aptamer-assisted assay, among others, represent rapid advances and emerging technologies in the area of non/semi/fully quantitative POC testing strategies [21]. Quantitative POC strategies such as microfluidics-powered lab-on-a-chip strategy can provide additional advantages over other POC methods such as multiplexing capabilities—combining antigen and antibody detection with high sensitivity and specificity, quantitation (such as RT-PCR or ELISA), ability to integrate smart algorithms, and intuitive reporting. While nucleic acid testing represents high sensitivity, evolving strains of SARS-CoV-2 often present false positive/negative testing, until such time that specific probes are available.

Whereas molecular diagnostic tests such as nucleic acid based testing including RT-PCR and antigen tests can only reveal whether a person is currently infected with SARS-CoV-2, antibody tests detect the body’s humoral immune response (IgG, IgA, IgM) following viral exposure and/or vaccination. Antibody responses can appear by day 5–7 post infection exposure, and it can persist in the bloodstream for many months after infection [22,23]. Serological assays can give insights into the host’s disease progression, vaccination response, and protective effectiveness [24]. Studies showed that IgG antibodies against viral proteins correlate with disease severity and outcomes [25,26]. Multiple SARS-CoV-2 vaccine studies demonstrated a direct correlation between vaccine efficacy, neutralizing antibody titers, and the titer of RBD targeting antibodies [24]. Thus, assessing anti-SARS-CoV-2 antibody titers can help inform vaccine efficacy and timing of booster administration, particularly in demand among at-risk populations where achieving vaccine-induced humoral protection was challenging [2,27]. Even in low-risk populations, protective efficacy wanes by 20–30% in the 6 months following vaccination [3], while newly evolved variants, such as the Omicron variants, showed that vaccination induced immune evasion [28,29]. These datasets informed the need for updating vaccination policy and monitoring vulnerable patient groups.

Molecular testing combined with serological assays can improve the overall diagnosis of SARS-CoV-2 [30,31]. However, current clinical settings that host concurrent antigen and antibody detection are limited due to need for expensive laboratory equipment, specialized technical training, and long assay wait times. A rapid and quantitative POC screening of SARS-CoV-2 viral antigen and viral-specific antibodies concurrently would certainly aid disease monitoring and management. The development and customization of these POC quantitative diagnostic tests tailored for the at-risk community (e.g., retirement homes, cancer care centers, critical care clinics, etc.) is a key priority alongside its use with gated patient screening and risk-based triage procedures. None of the existing diagnostic tests cover both the initial screening process as well as comprehensive POC diagnostic testing for those patients with elevated risks of infection. Over the past few years, we developed diagnostic tools suitable for POC clinical settings, including a platform to digitize biology with the capacity to learn [32], a COVID-19 seroprevalence assessment platform [33], an oral cytopathology platform for assessment of potentially malignant oral lesions [34]. Recently, we published a general framework for implementing a POC clinical decision support system [35] which was adapted to the task of predicting mortality in cardiac patients with COVID-19 [36]. More recently, a two-tiered system for evaluating COVID-19 prognosis in inpatient and outpatient settings was developed using data from a diverse population of patients across the New York City metropolitan area and externally validated using data from hospitals in Wuhan, China [31].

In the current study, we explored whether pre-screening patients using convenient non-laboratory data can predict COVID-19 status in patients without symptoms. This paper also demonstrated a quantitative strategy for concurrent COVID-19 and host antibody screening, suitable for use in POC settings, that has potential to be assisted simultaneously with the newly developed pre-screening method reported here. A preliminary assay validation was performed for this duplex COVID-19 test including a combination SARS-CoV-2 NP antigen and host IgG antibody, covering the entire diagnostic timeline of the disease with a single multiplexed test. This device integrated a lab-on-a-chip microfluidics platform facilitating automated liquid sample handling, with an easy, simple and sensitive assay readout and AI-assisted screening.

## 2. Materials and Methods

### 2.1. Patient Data

Pre-screening algorithms were developed from a retrospective analysis of asymptomatic or pre-symptomatic patient encounters resulting in a COVID-19 RT-PCR positive test. Data were collected across clinics and hospitals within the Family Health Centers (FHC) network at New York University (NYU) Langone from 1 January to 25 June 2020, although the first known positive case in the state of New York was detected 1 March 2020. Test positivity prior to this date was assumed 0. Data were analyzed at the encounter level rather than the patient level because many patients had multiple encounters. Symptomatic patient encounters, in which one or more primary COVID-19 symptoms (cough, fever, shortness of breath) was present, were excluded. Physiological predictors were evaluated at two levels (systolic blood pressure < 120 mmHg, diastolic blood pressure < 80 mmHg, body temperature ≥ 99 °F, pulse rate < 80 bpm, oxygen saturation ≤ 96%). County-level testing data were acquired from the New York State Department of Health (New York State Statewide COVID-19 Testing 2020). For each patient, a local positivity rate was calculated (i.e., the average test positivity rate within the county of the reporting health center from 8 days to 1 day prior to the patient encounter). Similarly, case incidence rate was calculated as the local 7-day average cases per 100,000. Consistent with NYU’s institutional review board (IRB) policy and federal regulations, this study did not involve human participants and did not require IRB review. The data set is available from the authors upon reasonable request and with permission of FHC at NYU Langone. Currently, with the changing dynamics of COVID pandemic scenario and infectivity across society, the COVID community clinics are no longer performing community surveillance data collection.

### 2.2. Model Development and Statistical Analysis

Pre-screening models were developed using similar procedures described in an earlier publication [37]. A lasso logistic regression model was trained to distinguish between asymptomatic or pre-symptomatic patient encounters that resulted in a positive vs. negative result for SARS-CoV-2 by RT-PCR. Continuous predictors were standardized with mean of zero and variance of one. Missing data were imputed using the multivariate imputation by chained equations package in statistical software R [38]. Samples in the training and test sets were partitioned and trained using stratified 5-fold cross-validation. Model cutoffs were selected to obtain at least 90% sensitivity. Diagnostic performance was documented in terms of mean area under the curve (AUC), sensitivity, specificity, positive predictive value (PPV), and negative predictive value (NPV). Normally distributed predictors were compared using an independent sample *t*-test. Proportions were compared using the chi-squared test [39,40]. Two-sided tests were considered statistically significant for *p* < 0.05.

### 2.3. COVID-19 Antigen/Antibody Assay Development

The quantitative POC antigen/antibody combination test was developed for the detection of SARS-CoV-2 NP and anti-S RBD IgG antibody. In-house fabricated agarose beads sensors, with potential to host a variety of proteins and molecules, were utilized as the backbone for assay chemistry. The anti-NP monoclonal antibody (Sino Biological, Wayne, PA, USA; #40143-R019) was conjugated to the agarose bead sensors, as was recombinant RBD protein. The RBD was produced in Expi293F cells transfected with the vector pCAGGS SARS-CoV-2 RBD (BEI Resources #NR-52309) following the methods of [41], but using PEI as the transfection reagent, then supplementing the media with valproic acid [42]. Anti-NP polyclonal antibody (Sino Biological #40588-T62) was conjugated to a fluorescent tag (Alexa Fluor 488 conjugation labeling kit, Invitrogen, Waltham, MA, USA; #A20181), and a secondary anti-rabbit antibody (Invitrogen) was also procured. Antigen (2019-nCoV nucleocapsid His recombinant protein, Sino Biological #40588-V08B) and antibody (2019-nCoV spike S1 antibody IgG, Sino Biological #40150-R007) assessments were made in PBS (Thermo Fisher Scientific, Waltham, MA, USA). A 10% (*w*/*v*) bovine serum albumin (BSA) (Sigma-Aldrich, St. Louis, MO, USA) solution was used for reagent stability, blocking nonspecific binding, and was used as sample carrier spiked in a dose-dependent manner with the analytes.

The assay design, reagent optimization, and proof of concept experimentation were performed on multi-well plates with inserts (Corning™ Trans well™ Multiple Well Plate with Permeable Polycarbonate Membrane Inserts, Fisher Scientific, Hampton, NH, USA). The polycarbonate membrane inserts allowed easy placement and immersion of the agarose beads in the sample buffer to allow the completion of different assay steps, somewhat mimicking the microfluidic environment within the cartridge, without disruption to the agarose bead integrity. Post wash steps, the beads were imaged under the fluorescence microscope by separating the inserts onto the imaging tray, while the beads were continued to be supported by the insert membrane. After the proof of concept and reagent optimization was completed, the assay was performed in a titrated manner with appropriate controls. Images were captured on a fluorescent microscope on FITC, Cy5, and DAPI channels and stacked to generate image outputs used for analysis, followed by whole bead fluorescence measurements. The concentration vs. intensity curve determined an initial detection range.

This concurrent NP antigen and host IgG antibody detection assay was next performed using prototype microfluidic cartridges adapted to the current application, non-form factor instrumentation, and software described previously [43]. The disposable injection-molded cartridge system was layered with the double-sided adhesive and polyethylene terephthalate laminates on top and a hydrophilic laminate material on the bottom allowed sample wicking through capillary action, and it consisted of interconnected microfluidics segments for facilitating various immunoassay stages, including sample and reagent introduction and delivery, mixing, bubbles and debris removal, and dedicated fluorescent analyte image acquisition through optical fluorescence signal readout. The fabrication of microfluidic cartridge, imaging, and analysis work was detailed in our recent publication, in development of our parallel effort to demonstrate the seroprevalence assessment of COVID-19 and vaccination-induced humoral antibody response, leading to this expanded work [33].

Analyte-specific beads were deposited into the cartridge, allowing multiple measurements on the same assay. The 16 min assay was performed at room temperature under continuous flow (PBS). Bead sensor priming, sample delivery, reagent incubation, wash steps, and image collection were completed using an Olympus fluorescent microscope and syringe pumps. Furthermore, standard curves for the concurrent assays were completed using spiked samples (0, 2.4, 10, 40, 160, 625, 2500, 2500, and 10,000 ng/mL) and fit to 5-parameter logistic regression. Limit-of-detection (LOD) values were calculated using blank control replicates (average signal intensity plus 3 standard deviations).

## 3. Results and Discussion

Evolving strains of SARS-CoV-2 continue to present high rates of transmissibility, infectivity, and an ability to evade vaccine-induced protection. The risk for severe disease remains high among at-risk individuals with high viral exposure, those with underlying medical conditions, and older adults. Although a large majority of the population received at least the primary series of vaccination and/or were infected at least once, the seroprevalence rates waned over time. With asymptomatic and pre-symptomatic cases serving as the main driving force for community spread, there remains concern that screening individuals for symptoms, elevated temperature, or vaccination record may be inadequate to detect subclinical infection and/or predict immune status. Concurrent infection and seroprevalence screening can help assess vaccination status, predict protection, manage patient care and risks, and monitor disease outbreaks.

**Figure 1:**
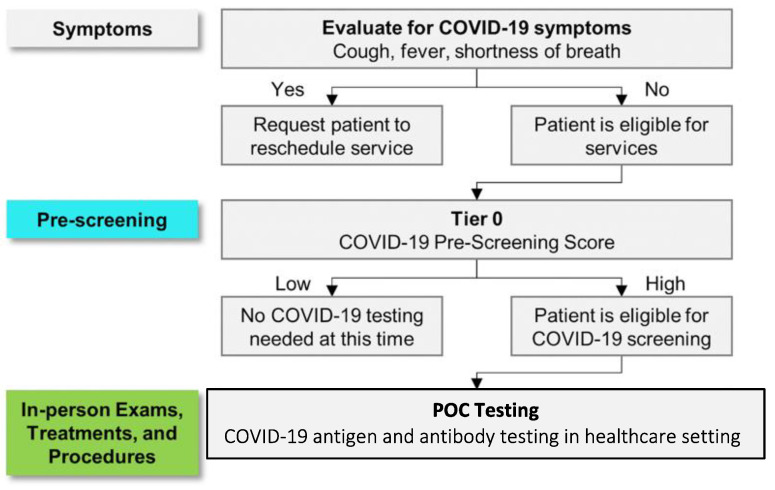
Clinical decision support system for COVID-19 screening. Prior to entering the care setting, patients may be screened for the presence of one or more COVID-19 symptoms (fever, cough, and shortness of breath). If symptomatic, patients should be rescheduled for a later date. The Pre-screening Algorithm (Tier 0) helps determine if a patient is eligible for COVID-19 screening. Patients with a high pre-screening score are recommended for the rapid antigen/antibody screening. Beyond the scope of this work and published elsewhere are prognostic models (Tier 1 and Tier 2) for predicting COVID-19 mortality in inpatient, outpatient, and hospital settings [36].

The novel screening approach described in this study can provide near real-time COVID-19 status, thus promptly identifying infected individuals and thereby reducing the risk of them spreading COVID-19. This tool can also screen individuals at high risk for developing severe disease and requiring hospitalization. This current study encompassed the development of an integrated COVID-19 screening capability for POC settings that fits within the scope of a larger multi-tiered clinical decision support ecosystem to assess the entire disease spectrum of COVID-19 in multiple care settings (Figure 1) [35]. One envisioned use of the proposed COVID-19 screening tool is for care providers in assessing the risk of transmission during in-person exams, treatments, or procedures. The process starts with patients seeking care, e.g., at an elderly care home, cancer care clinic, dental office, etc. Patients may be evaluated for the presence of one or more COVID-19 symptoms (fever, cough, and shortness of breath). If symptomatic, patients should be asked to reschedule for another date after their symptoms resolve. Patients without symptoms were then pre-screened using the pre-screening algorithm. Those with pre-screening scores above the high-risk threshold may be recommended for the rapid, quantitative COVID-19 antigen/antibody test.

A retrospective analysis determined whether pre-screening could effectively rule out COVID-19 negative patients (i.e., to reduce the number of unnecessary tests). Many patients had multiple encounters, and we based our analysis on a total of 3477 patient encounters resulting in RT-PCR tests at NYU Langone Health FHCs. Patient encounters with one or more primary symptoms (cough, fever, shortness of breath) were excluded (n = 924 encounters). The remaining 2553 asymptomatic or pre-symptomatic patient encounters either tested negative (n = 2059 encounters) or positive (n = 494 encounters) for SARS-CoV-2 by RT-PCR (Figure S1).

Table 1 shows the characteristics of the study population at the patient and encounter levels. A total of 1074 asymptomatic or pre-symptomatic patients were included. Comparing patients who tested positive vs. negative, age, gender, and body mass index were statistically similar. White and Asian populations accounted for a smaller proportion of the positives relative to those testing negative (*p* = 0.005 and 0.021). Those with Hispanic ethnicity accounted for 56.6% of the positives vs. 38.7% negatives (*p* < 0.001). Comorbid condition rates were similar in those that tested positive vs. negative. At the patient encounter level, all physiological measurements discriminated between the RT-PCR-positive and negative groups at their respective cutoffs (all *p* < 0.05). The local positivity rate was higher for patients testing positive (32.8%) vs. negative (17.7%) (*p* < 0.001). Similarly, the local case incidence rate was higher for COVID-19 positives vs. negatives (30.1 vs. 21.4 cases per 100,000, *p* < 0.001). Details of daily changes in positivity rates and case incidence rates from New York State Department of Health are detailed in Figure S2, though the models developed in this study used a 7-day averaged rate prior to the patient’s encounter.

**Figure 2:**
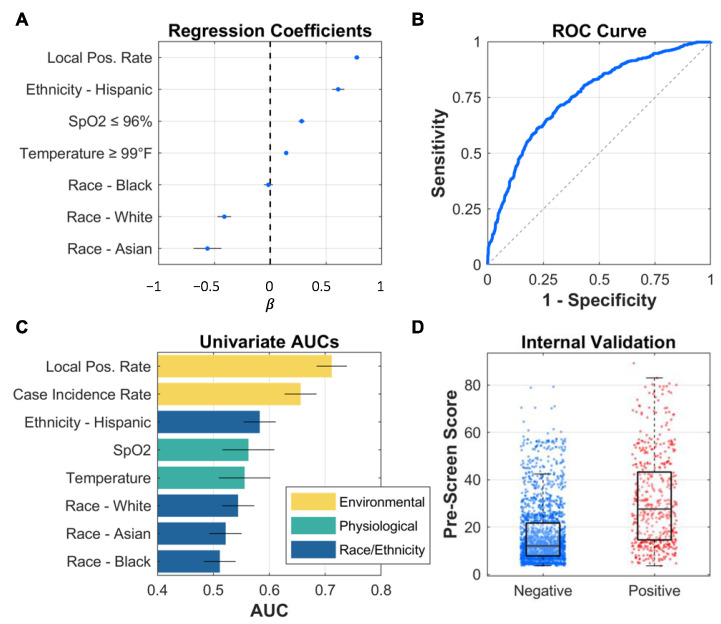
Model development results showing lasso logistic regression coefficients for the full model with local positivity rate, temperature, SpO2, race, and ethnicity (**A**), receiver operating characteristic (ROC) curve for the same model (**B**), univariate AUC values for predictors categorized by predictor type (environmental, physiological, race/ethnicity, and combination) (**C**), and box/scatter plot of the resulting scores from internal validation (**D**).

Pre-screening models for COVID-19 were developed and internally validated (Figure 2). The local test positivity rate was the strongest individual predictor (univariate AUC [95% CI] 0.71 [0.67–0.73]). The full model, which combined environmental, physiological, and demographic factors, had an AUC of 0.76 (0.73–0.78). Median (IQR) COVID-19 pre-screening scores were 12 (8–22) and 28 (15–44) for negative and positive patients, respectively.

**Figure 3:**
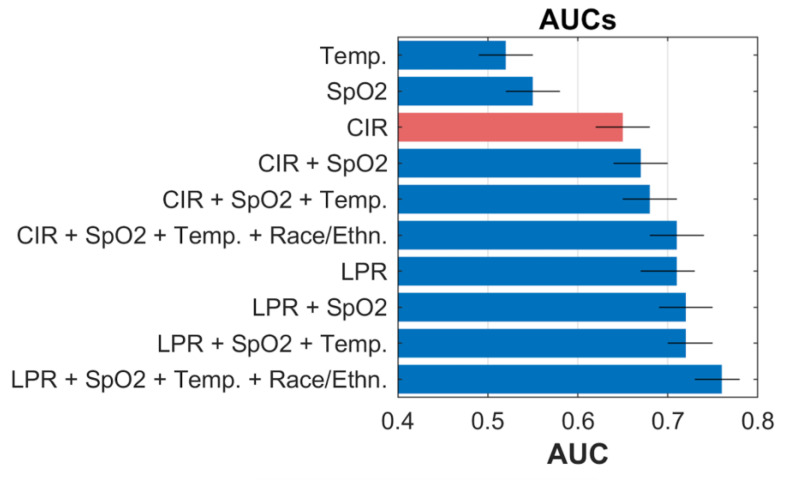
Diagnostic models for discriminating COVID-19 positive vs. negative (RT-PCR) in asymptomatic/pre-symptomatic individuals. The CIR-only model is the preferred pre-screening model (red). Temp. is body temperature ≥99 °F. SpO2 is oxygen saturation ≤96%. CIR is the case incidence rate. LPR is the local positivity rate. Race/Ethn is race and ethnicity of the 2553 patient encounters utilized for developing a pre-screening model in this study.

Figure 3 shows various diagnostic models that were developed to demonstrate the incremental effect of adding predictors, utilizing 2553 patient encounters (Figure S1). Despite being the default method for screening in clinical settings, temperature had only chance levels of association with PCR results (AUC = 0.52 [0.49–0.55]). The preferred model (case incidence rate only) had an AUC of 0.65 (0.62–0.68). The diagnostic performance of the full pre-screening model and the preferred pre-screening model, using case incidence rate, local positivity rate, oxygen saturation, temperature, race, ethnicity are shown in Tables S1 and S2.

Furthermore, the diagnostic performance for models discriminating COVID-19 positive vs. negative (RT-PCR) in pre- and asymptomatic individuals is demonstrated in Table S3. While the lasso regression coefficients for the full model and preferred model are stated in Tables S4 and S5.

Despite being the de facto method for COVID-19 screening to date, temperature was found to be relatively ineffective at distinguishing which pre- or asymptomatic patients were infected. However, temperature checks may still play an important role in detecting symptomatic individuals who unknowingly present with a fever. Likewise, measurements of oxygen saturation did not show significant improvements over temperature despite its potential importance in monitoring disease progression in confirmed COVID-19 cases. One unexpected finding of this analysis was that when and where a person was screened was the most important factor in predicting COVID-19 status. The local test positivity rate and case incidence rate were the strongest predictors of COVID-19 status, outperforming physiological and demographic factors. This result demonstrates the significance of time- and location-specific spread data within communities in estimating the pre-test probabilities for COVID-19 screening. This result may be especially relevant for large clinical centers which see an influx of patients from a broader geographic region.

Combining test positivity with recorded race and ethnicity changed the performance (AUC 0.76); however, the inclusion of racial and ethnic information was dependent on information collected at time of patient registration and may not generalize well to less diverse populations [44]. While comorbidities are widely recognized to increase risk of severe complications from COVID-19, there were no significant differences in BMI, diabetes, and renal disease between patients with/without positive RT-PCR. In addition, while test positivity rate was a better predictor than incidence rate, the testing data available to date are only reliably available at the US state level, not the county level, and are, thus, inappropriate for risk assessment in states with an uneven geographical distribution of cases. For these reasons, we designated the model with case incidence rate as the preferred model.

**Figure 4:**
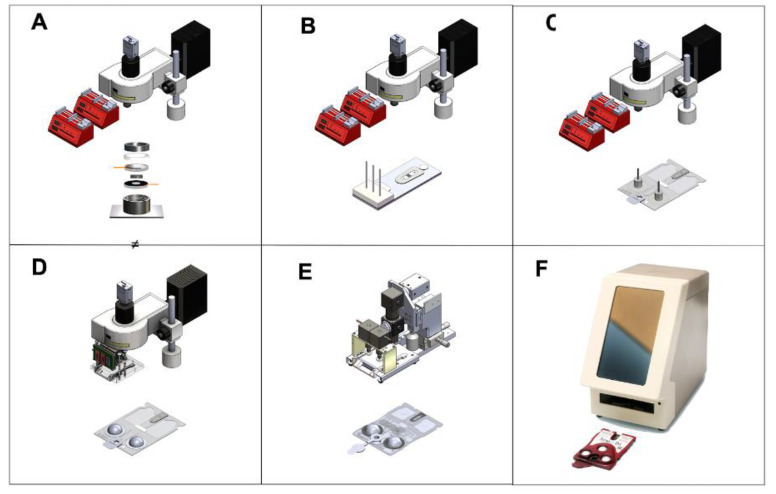
Cartridge and instrument evolution is shown for the following stages: (**A**) non form factor flow cell serviced with syringe pumps and imaged by commercial epi-fluorescence microscope, (**B**) non form factor laminate prototype serviced with syringe pumps and imaged by commercial epi-fluorescence microscope, (**C**) form factor laminate prototype serviced with syringe pumps and imaged by commercial epi-fluorescence microscope, (**D**) form factor laminate prototype with embedded blister packs and imaged by commercial epi-fluorescence microscope, (**E**) form factor laminate prototype with embedded blister packs and imaged by monorail customized epi-fluorescence image station, (**F**) production ready cartridge and analyzer instrumentation suitable for point of care measurements. The use of multiple stages of image instrumentation and cartridge has allowed for the various subsystems to be tested and key subcomponents to be isolated. At the time of this submission fully integrated instrumentation shown in panel F is available for drug testing applications. This instrumentation is designed to be programmable allowing for its adaptation to other applications including COVID-19 duplex testing. The application specific testing is planned for the near future.

While the pre-screening algorithm determined who is at elevated risk with 90% sensitivity (Table S2), the completion of COVID-19 antibody and antigen testing, which typically have high specificity (~99%), had the potential to improve the diagnostic performance. We envision that patients scoring above the threshold on the pre-screening assessment would be recommended for an on-site POC combinatorial antigen/antibody test. Assay validation on trans well plates with inserts permitted directed transition to optimization of assays through multiple stages of the microfluidics-facilitated assay on evolving cartridge and instrumentation designs, as detailed in Figure 4. The fully integrated microfluidic network enabled assay system shown in Figure 4F functioned as a portable diagnostic system applicable for POC testing.

**Figure 5:**
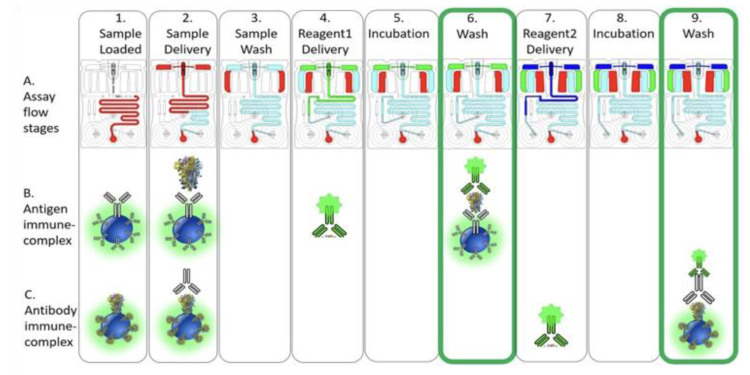
COVID-19 antigen/antibody assay sequence. Step 1 shows the sample (antigen +/− antibody) loaded to the cartridge input port, followed by sample delivery over the bead array through buffer flow via right blister (Step 2) and finished with a wash step (Step 3). Step 4 shows introduction of the antigen detection reagent conjugated to Alexa Fluor 488 (Step 4B) via the right reagent pad, over the bead array, followed by incubation (Step 5) and wash (Step 6) steps. In the presence of SARS-CoV-2 NP antigen in the sample, the post-assay completion image shows antigen capture beads fluorescing as a result of the antigen immune-complex formation (Step 6B). Finally, Step 7 shows the introduction of the antibody detection reagent conjugated to Alexa Fluor 488 (Step 7C) via the left reagent pad over the bead array, followed by final incubation (Step 8) and final wash (Step 9) steps. In the presence of SARS-CoV-2 IgG1 antibody in the sample, the post-assay completion image shows the antibody capture beads fluorescing as a result of the antibody immune-complex formation (Step 9C).

The conceptual basis for simultaneous COVID-19 NP antigen and host IgG antibody assay sequence is shown schematically in Figure 5. The antigen/antibody capturing beads helps complete the assay and capture the immunocomplex, while the presence of the control beads act as quality control simultaneously for each assay. First, during stage 1 (Figure 5), approximately 100 µL of the sample with/without antigen and antibody was introduced to the cartridge input port and simultaneously wicking through the long loop of microfluidic channel, short of the main bubble trap and vent membrane. During stage 2, the sample port was sealed once the sample is fully wicked into the channel and followed by enabling the sample delivery over the bead array through buffer flow via right blister. A standard transport protocol was developed through optimization of various priming, sample/reagent delivery, incubation, and wash flow rates and volume, using automated fluid routing to control buffer flow. In stage 3, following sample delivery, a wash step was enabled to remove unbound protein. Next, during stage 4, the antigen detection reagent (conjugated to Alexa Fluor 488) was introduced via the right reagent pad, over the bead array, through sustained buffer flow, followed by incubation, as seen in stage 5. The presence of the SARS-CoV-2 NP antigen in the sample, captured onto the beads followed by the detection reagent, causes the beads to fluoresce, as a result of the antigen immune-complex formation. Next, a thorough wash was enabled in stage 6. Finally, stage 6B shows the first image capture step, post wash, concluding the antigen detection immunoassay.

As the multiplexed immunoassay progressed towards the antibody detection steps, during stage 7, the antibody detection reagent (conjugated to Alexa Fluor 488) was introduced via the left reagent pad through buffer flow pushing the reagent over the bead array (Stage 7), followed by incubation (Stage 8) and final wash (Stage 9). In the presence of SARS-CoV-2 IgG antibody in the sample, the post-assay completion image (the second image captured during the entire assay) showed the antibody capture beads fluorescing as a result of the antibody immune-complex formation (Stage 9C).

This combination test modality allows for the placement of positive and negative control beads alongside the antigen and antibody reactive beads. Both antibody and antigen assays can be completed through the spatial placement of specific reactive bead ensembles within the array. Likewise, the 250–300 μm, in-house fabricated, analyte capturing agarose beads (conjugated with complementary protein) were placed in the 4 × 5 format bead array, each with a vertically tapering well.

**Figure 6:**
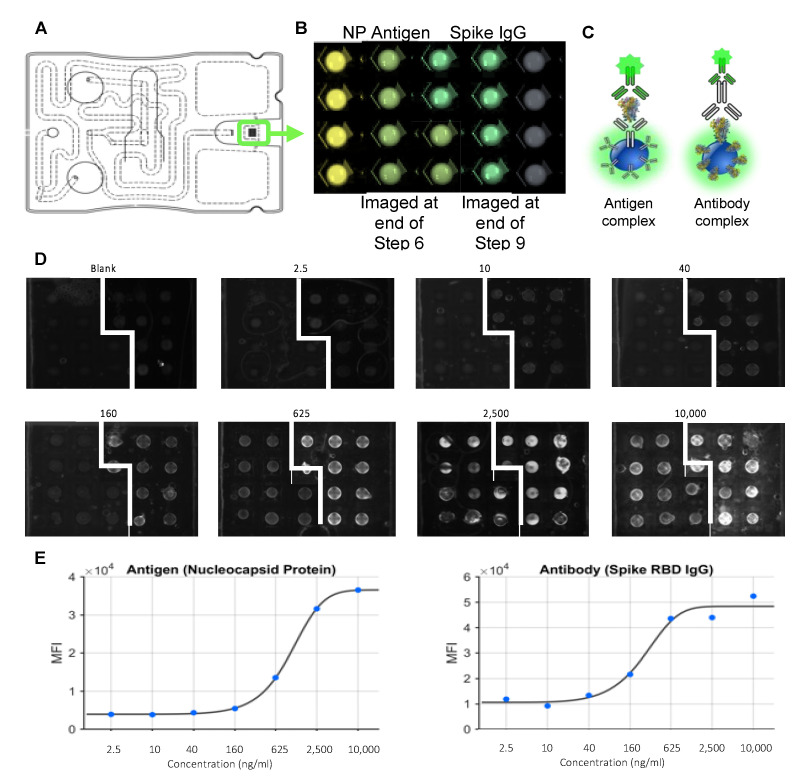
The POC microfluidics-based combination antigen/antibody assay tool. Illustration of assay cartridge (**A**) shows an array of 20 programmable agarose bead sensors (**B**), with antigen and antibody capture beads imaged separately at steps 6 and 9 of assay (see Figure 5 for sequence of fluidic steps), respectively, and stitched together to constitute the final image. The bead sensor serves as a high surface area substrate for developing programmable immunoassays for COVID-19 antigen and antibody detection (**C**). Multiplexed fluorescent images show bead sensor arrangement and captured analyte via fluorescence, with variation in signal intensity at various concentrations (**D**). Averaged bead fluorescence intensity (MFI) from the multiplexed assays were used to calibrate standard curves for the antigen and antibody tests (**E**). Standard curves were completed using spiked samples (0, 2.4, 10, 40, 160, 625, 2500, 2500, and 10,000 ng/mL) and fit to 5-parameter logistic regression. Limit-of-detection (LOD) values were calculated using blank control replicates (average signal intensity plus 3 standard deviations).

To demonstrate initial validation, standard curves for antigen (SARS-CoV-2 NP) and antibody (anti-S RBD IgG) were completed with 4-fold serially diluted analyte-spiked sample buffer, covering a range of high viral antigen and immune response load (10,000 ng/mL) to very low loads (2 ng/mL) (Figure 6). Standard curves showed a pattern of progressive fluorescence intensity and increasing signal-to-blank ratio (SBR), with intra-assay precision ranging from 7 to 25%. This work generated a LOD of ~24 ng/mL for the antigen and ~30 ng/mL for antibody detection. The LOD was determined over the five-parameter logistic regression fit, utilizing the calculated LOD MFI using blank control replicates (average signal intensity plus standard deviation) (Figure 6E). The clinical validation of this new AI integrated concurrent assay utilizing non-invasive sampling of saliva will be reported in future works.

Diagnostics at the POC are critical for successfully mitigating COVID-19 transmission risk in asymptomatic and pre-symptomatic populations. Expanding access to in situ testing capabilities adds significant convenience to the risk management infrastructure much needed in communities with vulnerable individuals, such as retirement homes, cancer care centers, and critical care clinics. While the current gold-standard RT-PCR and ELISA detection techniques are highly valuable, the added time, cost, and demand-supply chain are major bottlenecks for processing the growing needs for testing. Convenient antigen testing combined with rapid antibody-based testing has much potential in addressing these testing bottlenecks. In contrast to traditional immunochromatographic strip and ELISA techniques, the multiplexed microfluidics-based assay developed here has the potential to achieve high sensitivity in a convenient format with noninvasive sampling while maintaining high specificity.

Furthermore, viral antigen detection, in conjunction with screening host antibodies, may be a helpful strategy to achieve early detection of SARS-CoV-2 infection. NP, utilized in this work, is one of the most predominantly expressed and abundant protein in SARS-CoV-2 virions, ideal for early diagnostic detection, detectable up to 1 day before the appearance of clinical symptoms [45]. Importantly, it has three distinct domains, highly conserved across the coronaviruses, that are subject to lower mutagenic changes associated with newer evolving VOC, compared to the S and RBD protein [46,47,48], making them good targets for detection.

The concurrent assay developed here also utilized the anti-S RBD IgG to reflect the high utility and specificity of antibody-mediated protection associated with vaccination and/or infection. Most major COVID-19 vaccines elicit specific immune responses against the S protein of SARS-CoV-2, preventing the virion from host cell binding, fusion, and entry [49]. Additionally, SARS-CoV-2-infected individuals and/or vaccinated individuals both elicit a robust host IgG response against the S and receptor binding protein but not the NP [50]. This antibody-mediated response, particularly the IgG seroprevalence, was used as a surrogate marker to address the efficacy and protection conferred by the vaccines [48]. Thus, serological assays with IgG assessment can aid the understanding of a patient’s clinical status following viral infection and response to vaccination [24].

Our concurrent assay described a fully functional multiplexing of COVID-19 infection and host immune response biomarkers. A significant challenge with multiplexing is cross-reactivity between capture and detecting reagents, particularly in combining immunoassay formats. These issues can be mitigated through optimization of reagent sources, subtypes, blocking strategies, assay flow rates, and volumes. Additionally, limitations of this testing strategy included obtaining negative results in patients during their incubation period who later become infectious.

Multiple qualitative POC antigen or separately antibody detection strategies were approved by emergency used authorization by the FDA, significantly easing the testing bottlenecks across the pandemic timeline. Though the work in this manuscript represents a POC, quantitative, and concurrent antigen and antibody testing strategy, embodying a significant advancement over the antigen only or antibody only tests which are mostly non/semi-quantitative POC applications.

While this current work served to demonstrate initial method validation and a promising implementation for high-risk settings; requiring rapid, cost-effective, convenient, and accurate screening results, future work will involve further assessment of qualitative performance (sensitivity and specificity) and blinded validation of the combinatorial format with real patient samples confirmed by RT-PCR and lab-based serological testing methods.

A quantitative concurrent antigen/antibody screening platform may also be useful for assessment of COVID-19 convalescent plasma donor fitness. The passive transfer of anti–SARS-CoV-2 neutralizing antibodies from the plasma of recently recovered individuals to patients with severe COVID-19 is associated with a decrease in incidences of disease progression, hospitalization, and mortality [14]. Patients who are immunocompromised have higher risk for morbidity and mortality associated with COVID-19, since they less frequently elicit low antibody responses to vaccines. This convalescent plasma transfer therapy, when administered early in the disease course, is associated with mortality benefit for patients who are at high-risk for COVID-19 [14]. In these circumstances, early identification of a donor is key. This process can be aided by a rapid and accurate screening platform with concurrent antigen and antibody detection described in this paper, utilizing minimally invasive sampling such as saliva. Recently, another study showed the value of a concurrent antigen/antibody detection utilizing saliva [51]. Detecting SARS-CoV-2 from oro-/nasopharyngeal swabs requires high-quality specimens with a sufficient sampling of intact viral RNA. However, viral loads in the respiratory tract were shown to be highly variable, leading to high false-negative rates. Saliva emerged as a promising alternative to nasopharyngeal swabs for COVID-19 diagnosis and monitoring [52,53], in which testing accuracy may be improved by saliva’s more uniform availability of antigens and antibodies. The saliva sampling solution proposed here circumvents the limitations of oro- and nasopharyngeal sampling as patients can self-collect saliva samples with minimal instruction. Our work adds significant value over existing POC technologies, describing the incorporation of a pre-screening algorithm alongside a rapid, accurate, and quantitative, simultaneous SARS-CoV-2 NP antigen and host IgG antibody detection at the POC.

## 4. Conclusions

As the public health demands for COVID-19 pandemic change, there is a strong need to rapidly adapt testing strategies, monitoring both the infection and immune status of patients, especially directed towards the vulnerable populations. To facilitate public health policy decisions, governments across the globe use estimates of transmission rates, case numbers, and fatality rates. The assessment of infection can help mitigate transmission, while insights on virus-specific antibody titer levels can help recognize changes in the antibody-mediated protection affected by new, rapidly evolving SARS-CoV-2 variants and vaccination response. The pre-screening algorithm alongside simultaneous multiplexing capabilities and its streamlined workflow described in this paper represent important steps towards building an infrastructure necessary in this next phase of pandemic, continue protecting the at-risk individuals, and provide information to clinicians and public health policy makers to facilitate this process.

## Figures and Tables

**Table 1:** Characteristics of asymptomatic or pre-symptomatic patients resulting in a RT-PCR test for SARS-CoV-2 at NYU Langone Health’s FHCs. Data are represented as n (%) or mean ± standard deviation. chronic obstructive pulmonary disease (COPD). SpO2 is oxygen saturation. Local positivity rate is the 7-day average test positivity in the county where the patient received care. Local case incidence rate is the 7-day average case incidence in the county where the patient received care.

	RT-PCR Negative	RT-PCR Positive	*p*-Value
Patient-level			
No. of patients	770	304	
Encounters per patient	1.3 ± 0.6	1.2 ± 0.5	0.015
Age	48 ± 17	47 ± 17	0.443
Gender (no. of males)	280 (36.4)	112 (36.8)	0.883
Body mass index	29.3 ± 7.9	27.9 ± 5.3	0.130
Race			
White	298 (38.7)	90 (29.6)	0.005
Black	137 (17.8)	44 (14.5)	0.191
Asian	77 (10.0)	17 (5.6)	0.021
Other	258 (33.5)	153 (50.3)	<0.001
Ethnicity—Hispanic	298 (38.7)	172 (56.6)	<0.001
Cardiac comorbidities	218 (28.3)	73 (24.0)	0.154
Hypertension	186 (24.2)	70 (23.0)	0.696
Peripheral vascular disease	83 (10.8)	23 (7.6)	0.112
Heart failure	38 (4.9)	11 (3.6)	0.352
Cerebrovascular disease	30 (3.9)	14 (4.6)	0.598
Myocardial infarction	21 (2.7)	8 (2.6)	0.931
Ischemic heart disease	8 (1.0)	6 (2.0)	0.224
Asthma	81 (10.5)	24 (7.9)	0.192
Cancer	49 (6.4)	18 (5.9)	0.787
COPD	104 (13.5)	30 (9.9)	0.104
Diabetes	116 (15.1)	49 (16.1)	0.666
HIV/AIDS	4 (0.5)	3 (1.0)	0.391
Liver disease	30 (3.9)	12 (3.9)	0.969
Renal disease	35 (4.5)	13 (4.3)	0.848
**Encounter level**			
No. of encounters	2059	494	
Systolic blood pressure < 120 mmHg	270 (13.1)	141 (28.5)	<0.001
Diastolic blood pressure < 80 mmHg	426 (20.7)	186 (37.7)	<0.001
Temperature ≥ 99 °F	47 (2.3)	29 (5.9)	<0.001
Pulse < 80 bpm	251 (12.2)	87 (17.6)	0.001
SpO2 ≤ 96%	105 (5.1)	74 (15.0)	<0.001
Local Positivity Rate (%)	17.7 ± 17.6	32.8 ± 20.1	<0.001
Local Case Incidence Rate (cases per 100,000)	21.4 ± 15.8	30.1 ± 16.2	<0.001

## Data Availability

Data was generated by developing a novel detection platform at the McDevitt Lab. All data in support of this study is available by contacting the corresponding author and/or the first authors.

## Supplementary Materials

The following supporting information can be downloaded at: https://www.mdpi.com/article/10.3390/bioengineering10060670/s1, Figure S1: Disposition of patient encounters; Figure S2: Test positivity rates (A) and case incidence rates (B) from New York State Department of Health for the three counties in which the NYU Family Health Centers are located. While the figures below show daily changes in positivity and incidence, the models developed in this study used 7-day averaged rates prior to the patient’s encounter (i.e., averaged 1–8 days before encounter). Although, the dataset compilation from NYU Langone began in 1 January 2020, the first known positive COVID-19 case in the State of New York was detected on 1 March 2020, represented in this figure.; Table S1: Diagnostic performance of the full model (local positivity rate, SpO2 ≤ 96%, temperature ≥ 99 °F, race, and ethnicity); Table S2: Diagnostic performance of the preferred model (case incidence rate); Table S3: Table of diagnostic performance for models discriminating COVID-19 positive vs. negative (RT-PCR) in pre- and asymptomatic individuals. This table corresponds to the data shown in Figure 3 in the main text. Temperature is body temperature ≥99 °F. SpO2 is oxygen saturation ≤96%. CIR is the case incidence rate. LPR is the local positivity rate; Table S4: Lasso logistic regression coefficients for the full model; Table S5: Lasso logistic regression coefficients for the preferred model.
