# Supplementary material for: Simultaneous Quantitative SARS-CoV-2 Antigen and Host Antibody Detection and Pre-Screening Strategy at the Point of Care
Source: Bioengineering (Basel). 2023 Jun 1;10(6):670. doi: 10.3390/bioengineering10060670 (PMC10295356; doi:10.3390/bioengineering10060670)
Supplement: Supplementary file 1 [file bioengineering-10-00670-s001.zip › bioengineering-2363776-supplementary.pdf]

An Integrated AI and Simultaneous SARS-CoV-2 Antigen and Host Antibody Screening Strategy at the Point of Care

Supplementary Materials

Table S1. Diagnostic performance of the full model (local positivity rate, SpO2 ≤ 96%, temperature ≥ 99°F, race, and ethnicity)

	**Full Model**
AUC	0.76 (0.73–0.78)
Sensitivity	0.90 (0.89–0.91)
Specificity	0.39 (0.37–0.41)
PPV	0.26 (0.24–0.28)
NPV	0.94 (0.93–0.95)

Table S2. Diagnostic performance of the preferred model (case incidence rate)

	**Preferred Model**
AUC	0.65 (0.62–0.68)
Sensitivity	0.90 (0.88–0.91)
Specificity	0.23 (0.21–0.25)
PPV	0.22 (0.20–0.23)
NPV	0.90 (0.89–0.91)

Table S3. Table of diagnostic performance for models discriminating COVID-19 positive vs. negative (RT-PCR) in pre- and asymptomatic individuals. This table corresponds to the data shown in Figure 3 in the main text. Temperature is body temperature ≥ 99°F. SpO2 is oxygen saturation ≤ 96%. CIR is the case incidence rate. LPR is the local positivity rate.

	**AUC (95% CI)**
Temperature only	0.52 (0.49–0.55)
SpO2 only	0.55 (0.52–0.58)

CIR only (preferred model)	0.65 (0.62–0.68)
CIR + SpO2	0.67 (0.64–0.70)
CIR + SpO2 + Temperature	0.68 (0.65–0.71)
CIR + SpO2 + Temperature + Race & Ethnicity	0.71 (0.68–0.74)
LPR only	0.71 (0.67–0.73)
LPR + SpO2	0.72 (0.69–0.75)
LPR + SpO2 + Temperature	0.72 (0.70–0.75)
LPR + SpO2 + Temperature + Race & Ethnicity (full model)	0.76 (0.73–0.78)

Table S4. Lasso logistic regression coefficients for the full model

**Predictor**	**β**
(intercept)	-1.7505
SpO2 ≤ 96%	0.2822
Temperature ≥ 99°F	0.1434
Ethnicity - Hispanic	0.6096
Race - White	-0.4115
Race - Asian	-0.5630
Race - Black	-0.0160
LPR	0.7767

Table S5. Lasso logistic regression coefficients for the preferred model

**Predictor**	**β**
(intercept)	-1.5113
CIR	0.5266

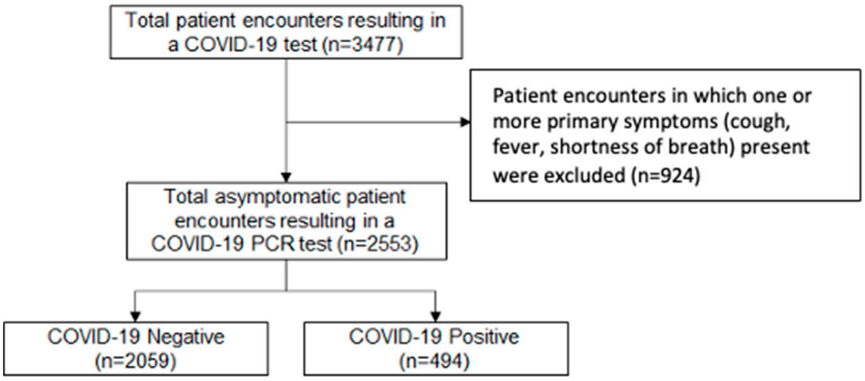

Figure S1. Disposition of patient encounters

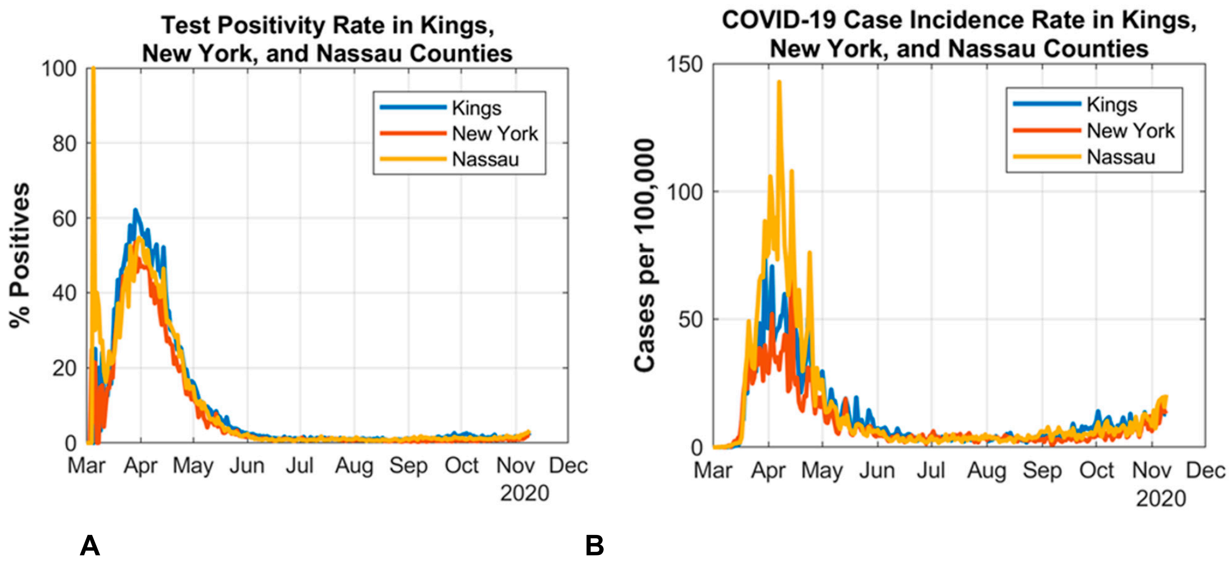

Figure S2. Test positivity rates (A) and case incidence rates (B) from New York State Department of Health for the three counties in which the NYU Family Health Centers are located. While the figures show daily changes in positivity and incidence, the models developed in this study used 7-day averaged rates prior to the patient's encounter (i.e, averaged 1-8 days before encounter). Although, the dataset compilation from NYU Langone began in January 1, 2020, the first known positive COVID-19 case in the State of New York was detected on March 1, 2020, represented in this figure.
